# How to change organisational culture: Action research in a South African public sector primary care facility

**DOI:** 10.4102/phcfm.v8i1.1184

**Published:** 2016-08-31

**Authors:** Robert Mash, Angela De Sa, Maria Christodoulou

**Affiliations:** 1 Division of Family Medicine and Primary Care, Stellenbosch University, South Africa; 2 Division of Family Medicine, University of Cape Town and Western Cape Department of Health, District Health Services, South Africa

## Abstract

**Background:**

Organisational culture is a key factor in both patient and staff experience of the healthcare services. Patient satisfaction, staff engagement and performance are related to this experience. The department of health in the Western Cape espouses a values-based culture characterised by caring, competence, accountability, integrity, responsiveness and respect. However, transformation of the existing culture is required to achieve this vision.

**Aim:**

To explore how to transform the organisational culture in line with the desired values.

**Setting:**

Retreat Community Health Centre, Cape Town, South Africa.

**Methods:**

Participatory action research with the leadership engaged with action and reflection over a period of 18 months. Change in the organisational culture was measured at baseline and after 18 months by means of a cultural values assessment (CVA) survey. The three key leaders at the health centre also completed a 360-degree leadership values assessment (LVA) and had 6 months of coaching.

**Results:**

Cultural entropy was reduced from 33 to 13% indicating significant transformation of organisational culture. The key driver of this transformation was change in the leadership style and functioning. Retreat health centre shifted from a culture that emphasised hierarchy, authority, command and control to one that established a greater sense of cohesion, shared vision, open communication, appreciation, respect, fairness and accountability.

**Conclusion:**

Transformation of organisational culture was possible through a participatory process that focused on the leadership style, communication and building relationships by means of CVA and feedback, 360-degree LVA, feedback and coaching and action learning in a co-operative inquiry group.

## Introduction

South Africa is in a process of strengthening primary healthcare in order to provide universal coverage and improve equity through the introduction of national health insurance.^1^ Organisational capacity at a sub-district level is needed to deliver on this aspiration.^2^ This organisational capacity has been conceptualised as ‘hardware’ and ‘software’.^2^ Hardware refers to the tangible infrastructure, finances, human resources and technology required. Software refers to the observable skills and competencies as well as the organisational systems and procedures. However, software also refers to the less visible values, norms, relationships, communication and use of power within the health system. These intangible aspects are the hidden drivers of the organisational culture, which is experienced by both patients and staff.

The healthcare workers are the most important resource in the health system. The effective functioning of an organisation not only depends on the competence and technical expertise of its workers but also on their job satisfaction, motivation, engagement and performance.^3^ Currently, high levels of burnout have been reported, which suggests that healthcare workers, however competent, may not have the emotional and personal resources to form effective patient-centred relationships.^4^ Healthcare workers are in fact frequently criticised for being abusive and rude.^5^ Patients may therefore experience an organisational culture that lacks caring, compassion, empathy and support.

At the same time, the staff experience of the organisational culture has been described as strongly hierarchical, with decision making dominated by command and control approaches implemented through organisational silos (of directorates and units), in which management is traditionally seen as an administrative function rather than a proactive process of enabling learning, and in which control is exercised in an authoritarian manner.^6^

Changing organisational culture has been identified as a key goal in improving the resilience and quality of a resource-constrained health system.^6^

The Western Cape Department of Health has emphasised that improving health outcomes, quality of care and patient experience is central to their vision.^7^ Part of their approach to achieving this is a more values-based approach within the organisation that identified caring, competence, accountability, integrity, responsiveness and respect (C^2^AIR^2^) as the core desired values.^7^ Nevertheless, recent cultural values assessment (CVA) have shown that the top 10 current organisational values in the metropolitan district health services included poor sharing of information, cost reduction, confusion, control, manipulation, blame, power, hierarchy and long hours.^8^ These results indicated that the current organisational culture was limiting staff performance and engagement.

The Negotiated Service Delivery Agreement between the President and the Minister of Health acknowledges the need to strengthen management at the facility level.^9^ Despite this, there has been very little research on the challenges of organisational transformation at the facility level in South Africa. Retreat Community Health Centre (CHC) was one of the facilities that participated in the CVA^8^ and after receiving their feedback expressed interest in a process to change their organisational culture. Therefore, the aim of this study was to explore how to transform the organisational culture in line with the desired values expressed in the baseline assessment and by the Department of Health.

## Methods

### Study design

A co-operative inquiry group (CIG) used participatory action research to engage with the question of how to transform organisational culture at Retreat CHC over a period of 18 months. Change in the organisational culture was measured at baseline and after 18 months by means of a CVA survey. The three key leaders at the health centres also completed a 360-degree leadership values assessment (LVA) and had 6 months of coaching based on the feedback.

### Setting

Retreat CHC serves the community of Retreat in the City of Cape Town. People living in Retreat are mostly from a low socio-economic background, usually speak either English or Afrikaans and historically belong to the so-called ‘coloured’ community. The CHC had a facility manager, family physician and nursing managers in charge of operations, trauma unit, HIV unit and maternity unit. There was also a pharmacy manager and an administrative officer in charge of reception. It offered ambulatory primary care services as well as 24-hour emergency care to both adults and children as well as 24-hour maternity services. A multidisciplinary team consisted of a family physician, medical officers, primary care nurses, midwives, pharmacists as well as allied health and dentistry staff. In addition, support services included cleaners, security guards, a porter, a personal assistant and clerks. Total staff complement at the facility totalled 128 people.

### Formation of co-operative inquiry group

The purpose of the study and CIG process was presented to a meeting of senior staff, invited by the facility manager, from all the departments at the CHC. Fourteen people consented to participate in the CIG and included the facility manager, family physician, five senior nursing managers, a medical officer, a post basic pharmacist’s assistant, nurses working in the preparation room, emergency centre and maternity unit, the supervisor of the general assistants and the radiologist. Towards the end of the CIG process, six additional staff members joined the CIG. These included a new pharmacy manager, a new administrative officer and four other staff who were leading the C^2^AIR^2^ club process. As the C^2^AIR^2^ club initiative also focused on promoting key organisational values, the management felt it made sense for them to join the CIG. The principal researcher, who was not a member of staff at Retreat CHC, facilitated the CIG.

### The co-operative inquiry group process

The CIG followed a cyclical process of planning, action, observation and reflection over a period of 18 months from June 2014 until November 2015. The meetings of the CIG are summarised in Table 1. At the initial meeting, the group reflected on the results of the baseline CVA survey. The researcher then conducted a focus group meeting with staff to make sense of the results of the survey and gave feedback on this at the second CIG meeting. By the third meeting, the group had identified the key issues that they wanted to focus on and planned initial actions to address these. At all subsequent meetings, the group gave feedback on the actions that they had attempted during the previous period, engaged in group reflections and activities to look deeper into the key issues that emerged and spent time planning individual and group actions for the next period. Group feedback and discussions were recorded on digital audio tapes and a summary created immediately after each meeting.

**TABLE 1:** Co-operative inquiry group meetings.

CIG	Date	Attendance	Focus of CIG meeting
1	13 June 2014	12	Interpretation of values in the Barrett’s survey for the current and desired culture
2	25 July 2014	12	Feedback on themes from the focus group interviews. Prioritisation of the key issues that need to be addressed using the nominal group technique: Improving open communication, reducing cultural entropy from cost reduction, improving relationships, improving accountability.
3	22 August 2014	13	Exploration of how to improve open communication and relationships Trust Matrix Exercise looking at strengths and weaknesses of the team relationships Mapping of the communication network and how information flows in the CHC Individual and collective plans for action
4	7 November 2014	11	Feedback and reflection on actions Exploration of how to reduce cultural entropy from cost reduction and improve accountability Envisioning the future – how people and groups in the CHC would behave, relate and perform if the desired culture was achieved Individual and collective plans for action
5	13 February 2015	11	Feedback and reflection on actions Scoring of progress towards the desired culture (values) Reflection on the scoring of the progress Individual and collective plans for action
6	17 April 2015	11	Feedback and reflection on actions Drawing of ecomaps reflecting quality of relationships Individual and collective plans for action
7	3 July 2015	11	Feedback and reflection on actions Thinking councils on ‘How do we make staff feel safe to give feedback to managers or departments?’ ’How do we co-ordinate our efforts to make achieving our goals as efficient as possible and minimise stress on individuals?’ Individual and collective plans for action
8	20 November 2015	9	Reflection on how the organisational culture had changed over the 18 months and construction of the consensus of learning using the nominal group technique.

CHC, Community Health Centre; CIG, co-operative inquiry group.

### Leadership values assessment and coaching

During June and July 2014, the three key leaders at the CHC (facility manager, family physician and nursing manager) also had 360-degree LVAs conducted. The LVA is said to be a 360-degree assessment because feedback is elicited from managers, colleagues and subordinates who work all around the leader. These LVAs give feedback on how these respondents experienced them as leaders in terms of their values and compared this assessment to their own perceptions. An independent personal coach gave them the feedback and then provided them with 6 sessions of individual coaching over a period of 6-months.

### Cultural values assessment survey

All staff at the CHC were invited to complete a CVA survey at baseline (January 2014) and follow-up 18 months later (August 2015). The CVA measured how staff perceived their personal values, the values expressed in the current organisational culture and the values that they would like to see in the future culture. The CVA is a validated tool produced by the Barrett’s Values Centre and offers the respondents a list of personal and organisational values to choose from.^3^ This tool was selected as it was already an accepted approach to measure organisational culture in the Government of the Western Cape and Department of Health. Values could be positive or limiting. Limiting values were ones that usually limit the performance of the organisational system. Each respondent selected their top 10 personal and current and desired organisational values. These were then collated and analysed by the Barrett’s Value Centre who provided a detailed report.

The CVA was used in two different ways in this study. Firstly, the baseline report on organisational culture was used to stimulate reflection and planning in the CIG as described above. An integral model of the organisation was used for this process of reflection.^3^ In this model, whole system change is based on improving the alignment between all four quadrants of the model shown in Table 2. Personal alignment refers to the extent to which people’s personal values are expressed in their professional behaviour as individuals in the CHC. Structural alignment refers to the extent to which the organisational values are expressed in the organisation’s structure, processes and procedures. Values alignment refers to the alignment of personal and organisational values and the extent to which people can bring their values to work. Mission alignment refers to the alignment between people’s personal behaviour and the organisational structure, processes and procedures and the extent to which people can engage with and commit to the organisational modus operandi.

**TABLE 2:** Four quadrants of human systems.

Variable	Internal	External
Individual	Personality: Individual values and beliefs	Character: Individual actions and behaviours
Collective	Culture: Collective values and beliefs	Social structures: Collective actions, behaviours and processes

*Source:* Barrett 2013

Secondly, the CVAs performed at baseline and 18 months later were used to compare the extent to which the organisational values had changed over this period. The degree of cultural entropy was also measured, which is the amount of energy in a group consumed in unproductive work and is a measure of the conflict, friction and frustration that exists within a group. It was measured as the percentage of all values selected that were limiting values.

Values could also be analysed as belonging to seven different levels of organisational consciousness as shown in Table 3, and this model was also used to reflect on the transformation required from current to desired organisational culture as well as to monitor the actual transformation over time.^3^ The top 10 organisational values were plotted according to these seven levels for both the current and desired culture at baseline and follow-up. The pattern that emerged indicated where the organisation was currently investing its energy and what organisational needs were being addressed. Levels without any values could represent a blind spot for the organisation that needed attention, a level that had been fully achieved and did not require attention or an opportunity for future development. A well-functioning organisation should have full-spectrum consciousness with values across all seven levels.

**TABLE 3:** Seven levels of organisational consciousness.

Level of consciousness	Example of positive collective values
7. Service: Self-less service to the world	Social responsibility, future generations, long-term perspective, ethics, compassion, humility
6. Making a difference: to the local community or health district	Collaboration, community involvement, strategic partnerships. Staff fulfilment, coaching/mentoring, leadership development
5. Internal cohesion: Building internal organisational community	Shared values, vision, commitment, integrity, trust, passion, creativity, openness, transparency
4. Transformation: Continuous renewal and learning	Accountability, adaptability, empowerment, teamwork, goals orientation, personal growth
3. Self-esteem: High performance and quality of care	Systems, processes, quality, best practices, pride in performance. *Bureaucracy, arrogance, image, hoarding information*
2. Relationships: With colleagues and patients	Loyalty, open communication, patient experience, friendship. *Blame, internal competition, rivalry, manipulation*
1. Survival: Resources and safety	Sufficient budget, equipment, employee health, safety. *Control, chaos, caution, job security*

*Source*: Barrett 2013

*Note*: Limiting values are shown in italics.

### Ethical considerations

The study received ethical approval from the Health Research Ethics Committee at Stellenbosch University (Ref N12/10/059) and permission from the Department of Health in the Western Cape.

## Results

### Organisational culture at baseline

**FIGURE 1:**
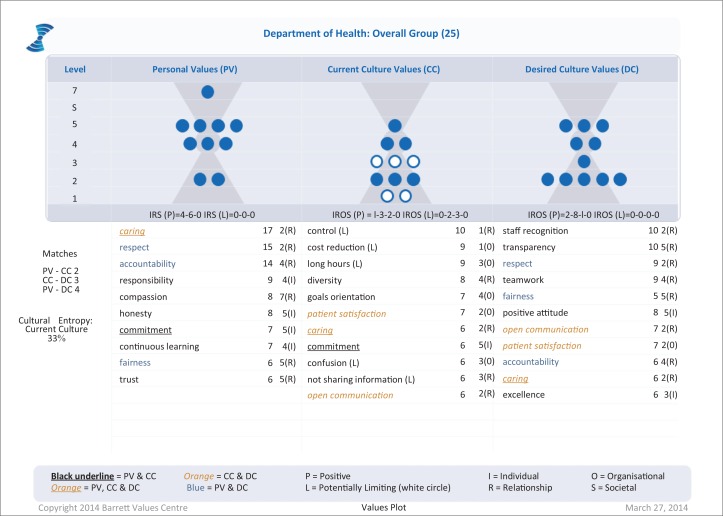
Baseline cultural values assessment.

Twenty-five staff members completed the baseline survey, including 5 nurses, 5 allied health professionals, 11 support staff and 4 unknown. The top 10 personal, current and desired organisational values are shown in Figure 1 and plotted against the seven levels of organisational consciousness. Five of the values in the current organisational culture were limiting values (control, cost reduction, long hours, confusion, not sharing information), which were likely to be reducing staff engagement and organisational performance. Overall levels of cultural entropy were high (33% of all values selected were limiting values), which according to the Barrett’s Centre report implied serious problems requiring cultural and structural transformation, leadership development and coaching. Caring was a value shared across all three domains, while commitment was the only other personal value found in the current culture. Personal values of respect, fairness and accountability were asked for in the desired culture. Patient satisfaction and open communication were present in both the current and desired culture. The desired culture asked for a shift of focus to levels 2 and 5 indicating a need to pay more attention to relationships, building a sense of community and shared purpose. The values at level 1 and 3 were hindering organisational performance, and staff asked for a focus on excellence instead of these values.

These results were presented to both the staff and CIG, and the final interpretation of these organisational values is given in Table 4.

**TABLE 4:** Interpretation of the selected values.

Value	Interpretation
Control	An autocratic top–down management style, which gives orders and makes rules, but is not open to communication or feedback. Too much control stifles individual freedom and choice (e.g. to help a colleague when necessary), as well as innovation (e.g. feeling safe to experiment), through a fear of letting people take control of their own work.
Cost reduction	A perception that staff are asked to achieve the same outcomes, with increasing workload, but with less resources (particularly human resources). The message received is that there is no money for locums, for supplies or equipment even though these may be essential to performing one’s role or function well. Essential resources are often missing.
Long hours	People are not necessarily working more hours than they should, but are often put under extreme pressure during their working hours such that the hours feel very long. For example, there may be no arrangements to provide extra cover during leave and the person left behind has to work twice as hard to cope with the same workload. Or you may be given a different volunteer to help you every day who does not know what to do.
Diversity	Both the staff and the patients come from a wide variety of different languages, professions, social and cultural backgrounds. One cannot treat or manage everyone the same.
Goals orientation	The health centre works towards goals and targets set and monitored by the District Health Services.
Patient satisfaction	Staff feel that they are here for the patients and most patients are satisfied. There is a long-standing commitment to patient satisfaction as a core value. Staff do receive verbal expressions of appreciation and sometimes small gifts. Management often focuses on complaints, which then dominate the picture. The dissatisfied patients are more vocal, although complaints are often justified regarding long waiting times, missing folders and rude staff.
Caring	Staff feel they are here to care for the patients and look after those less fortunate than themselves. Staff feel that overall there is a culture of caring for the patients, but not for the staff. Management should see the workforce as a family and not a machine. Managers should show more appreciation, responsiveness, concern and compassion for the staff.
Commitment	Staff feel that they are committed to quality care and efficiency. Whether you are a receptionist, nurse or doctor you have a specific role or contribution and a commitment to deliver on this. The level of commitment of staff to their work depends on how cared for they feel by the organisation and is also reflected in the amount of teamwork and engagement.
Confusion	Not sharing information leads to confusion, especially for the patients who do not know how to access services, where to go or why their expectations are not being met. Staff receive different instructions from different managers and may misinterpret information that is poorly communicated. Diversity also contributes to different interpretations. New staff are not well orientated to the work, which leads to confusion as to what is expected. People are thrown into the work as staff shortages and the pressure to offer the service leaves little space for proper orientation.
Not sharing information	The CHC has started to improve the sharing of information with patients, but many patients do not know what is happening. Staff do not know how each other’s services are organised and so may also share wrong or conflicting information with patients. Need to understand how the whole system works so patients can be informed correctly. Information is also not always shared or not shared well within the consultations. Patients leave the consulting room confused or misunderstanding what was said and this relates to poor compliance and control. Communication should be open and honest. What people say and what they do should be congruent, which also links to accountability. Miscommunication is a problem. Reasons for meetings are not always clear and then people decide not to come and rely on feedback from others, which is often inaccurate. Staff meetings have no agenda or minutes taken; therefore, what is decided is not clearly defined or followed up on. Communication is often one way – what is concerning the management only. There is also little sharing of information from management meetings that occur higher up the hierarchy. Decisions not always shared across the whole staff; for example, some groups are told they can go early and others not.
Open communication	It is interesting that both ‘not sharing information’ and ‘open communication’ were selected as they appear contradictory. The CIG thought that ‘not sharing information’ was most likely the view of staff at the bottom of the hierarchy, while ‘open communication’ the view of staff in leadership positions. The multidisciplinary team meetings have improved open communication. There needs to be more open communication when there are problems such as not receiving ones salary on time. Show respect and be more transparent about what is going on rather than staying quiet. The staff feel that positive communication will improve staff morale thus rendering excellent service delivery in turn. Staff hold a lot of hurt and resentment and do not feel they can share their feelings.
Staff recognition	Show more recognition for what staff are doing, which goes beyond just counting numbers of patients seen. Some staff feel that they are isolated and not valued. Recognise the efforts of staff to go beyond what is required because of leave and shortages of staff. This does not necessarily imply a structural response (i.e. incentives, rewards) but an attitude of appreciation for staff that go the extra mile, or an ability to hear the feelings and emotions. Often made to feel like you are moaning and complaining, or not showing enough respect for the authority of managers.
Transparency	Transparency is linked to open communication and respect. Need to be more transparent about the budget and why it takes so long to solve problems related to providing better healthcare. Things take forever to fix or order. However, there is a perception that the management get what they need more easily, such as computers and printers. Suspicion that resources are not allocated fairly.
Respect	Applies to both patients and staff. Speak to each other as colleagues and professionals, how things are communicated is important. Respect is shown by being responsive to staff concerns (e.g. safety and security) and requests as well as sensitive to giving feedback without publically ordering, blaming or shaming people. Develop kind and considerate interactions, reduce gossip. Respect patient’s viewpoints and take proper consent for procedures. Sometimes, patients lack respect for the staff and swear or are rude, especially in reception.
Teamwork	Staff work together to help patients flow through the system and access care. Need to reduce confusion by better teamwork in which everyone understands how the health centre is operating and what the problems are on a daily basis. Breakdown silos between departments so that all know how others are working and can also better inform patients on how to navigate the services. Need to reduce the separation between management and staff ‘on the floor’ and the perception that management are unsupportive and controlling.
Fairness	What counts for one, should count for all. Staff should be held accountable for a fair contribution to the workload and not reprimanded for failing to meet unreasonable expectations. Better communication and more transparency will reduce perceptions of unfairness in the way people and departments are treated.
Positive attitude	People hope for a more engaged, committed and appreciative staff, with a ‘can do’ attitude.
Accountability	Staff should be held accountable for the consequences of their actions. For example, the pharmacy often takes the brunt of complaints and unhappy patients because of things that have happened during the patient’s journey through the centre. If you say something, actually do it. When things go wrong, people are not held accountable or do not take responsibility.
Excellence	People feel they are striving for excellence despite the problems, but are often not recognised for going the extra mile.

CHC, Community Health Centre.

Following the feedback described above, the CIG summarised the key issues as improving communication, building relationships, reducing the perception of cost reduction as a driving force and increasing accountability.

### Organisational culture at follow-up

**FIGURE 2:**
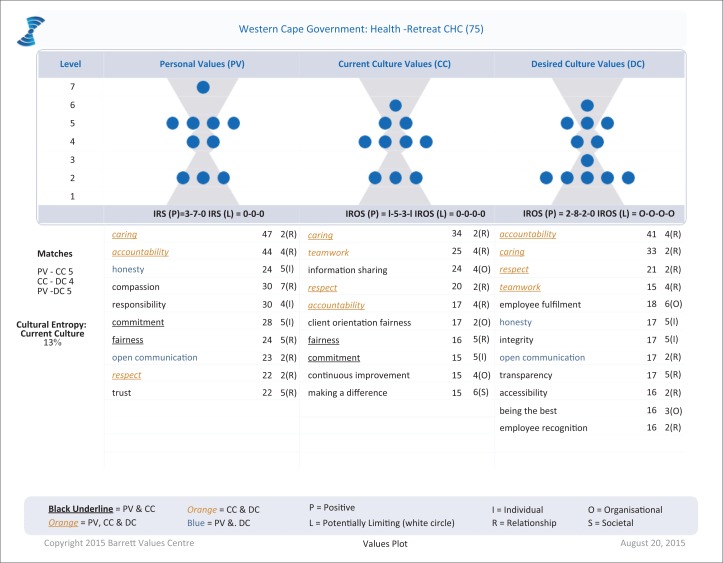
Follow-up cultural values assessment.

The follow-up CVA was completed by 75 staff members, including 5 doctors, 41 nurses, 7 allied health professionals and 22 support staff. The results are shown in Figure 2.

At follow-up there were no limiting values in the top 10 values and cultural entropy had dropped from 33 to 13%. Caring had moved to the top value experienced at the health centre, and all three personal values that were previously asked for as part of the desired culture (respect, fairness and accountability) were now recognised as part of the current culture. Teamwork, making a difference and continuous improvement were also recognised in the current culture and mirrored the values of teamwork, positive attitude and excellence that were also previously asked for in the desired culture. Information sharing was now an unequivocal part of the organisational culture suggesting that communication was improving. Patient orientation had replaced goals orientation in the current culture.

The concentration of values in the current culture at level 4 suggested a focus on organisational transformation and an emerging ability to deliver on this. In the desired culture, staff continued to ask for a greater focus on relationships (level 2), shared community and purpose (level 5). There was still a need to make accountability, staff recognition and fulfilment a greater part of the current culture. More open communication, transparency and accessibility were aspired to, even though information was more shared. Staff remained committed to excellence and being the best.

### Strategies used to transform organisational culture

The CIG reached a consensus on the activities that were responsible for transforming organisational culture and ranked them as shown in Table 5.

**TABLE 5:** Strategies used to change organisational culture.

Rank	Score	Key learning
1	20	Leadership coaching to help leaders develop
2	15	Change in management style from authoritarian/telling to more collaborative/listening
3	13	Being personally more open to people and approachable
3	13	Change in way meetings were run: more interaction, eliciting feedback, respect people’s opinions, responsive, better documented, more accountable for decisions made
4	8	Creating more effective teams/groups in the CHC that communicate regularly, look after each other, prioritise and plan
4	8	Management team more open about the strengths and weaknesses of the organisation and more vulnerable about their own personal strengths and weaknesses
5	7	The co-operative inquiry group process and external facilitation
5	7	Giving change enough time to happen
6	5	Commit time to the CIG meetings and process
6	5	Management team welcoming feedback, for example, book for suggestions/complaints, eliciting feedback in staff meeting
6	5	Leadership seeing that organisational culture is an important issue and being willing to engage with it
7	4	Sharing what each department is doing with all the staff – being aware of the contribution and role of other staff
7	4	Delegating ‘micro’ responsibilities to specific people within the team, for example, doctor’s group
8	3	C^2^AIR^2^ club groups (x6 – one for each key value) that involve all staff and all departments in each group
9	2	Having regular/monthly social activities or events for the staff
10	1	Empower mid-level managers more, for example, take more responsibility in staff meeting, staff members following the right channels
11	0	Improve communication via WhatsApp groups and notice boards

CHC, Community Health Centre; CIG, co-operative inquiry group; C^2^AIR^2^, responsiveness and respect.

A large number of the strategies involved changing the managers’ leadership style through their willingness to engage with the issue of organisational culture, personal coaching and involvement in the CIG. Managers became more confident, open, vulnerable, approachable, collaborative, appreciative and empathic. Small changes in their approach to leadership led to a large change in the way the organisational culture was perceived. Managers felt that the process illuminated key aspects of the organisation that had not previously been recognised as important. The coaching process also led to greater bonding and teamwork between the managers.

Communication of information and decisions were improved as well as opportunities for feedback from staff to managers. Relationships between staff members and teamwork were improved by ensuring that different departments understood each other’s functioning, social events, social media and delegation of responsibility within teams. The action research process itself and the C^2^AIR^2^ club groups were recognised as important parts of the process of change.

## Discussion

### Key findings

The study affirmed that despite the many resource constraints and workload challenges in the public sector, organisational transformation is possible at the level of the primary care facility where the majority of patients and health problems are seen.

Cultural entropy decreased dramatically, indicating that the organisation was functioning better, although entropy should still be reduced further to less than 10% for healthy functioning.^3^ The key drivers of this transformation were change in the leadership style and functioning through feedback from the CVA and LVA, personal coaching of the three key leaders and engagement of broader leadership in the CIG process.

Retreat CHC moved from a culture that emphasised hierarchy, authority, command and control to one that established a greater sense of cohesion, shared vision, communication, appreciation, respect, fairness and accountability. Trusting the majority of your professional staff to strive for excellence, to innovate and to care for patients instead of fearing that you needed to police, blame or manipulate them into delivering on goals was a fundamental shift. The willingness of leaders to engage with these issues and allow small changes in their approach to leadership led to substantial improvement.

### Discussion in relationship to literature and policy

The study took a values-based approach to understanding organisational culture and leadership. Organisations often espouse a set of values, without the ability to actually embody them ‘on the floor’.^3^ This study has demonstrated how values can be engaged with practically to drive change. The integral model outlined in the methods section was a key conceptual framework to make sense of the links between personal and collective values as well as individual behaviour and collective processes and procedures. The focus group interviews, which contextualised the meaning of these values, were also a key part of grounding these values in reality.

The study also confirmed that changing the leadership style was the key factor in enabling this transformation. Managers at Retreat CHC came to embody the concept of managers who led rather than just administered.^6^ Managers who lead have been characterised as focusing on collaborative actions taken by groups, seeing the possibilities to make things better, taking responsibility and initiative to tackle challenges, focusing on activities that are aligned with results that matter, displaying generosity and concern to serve the common good and inspiring others to do the same.^6^ The organisational culture is largely created by the type of leadership; therefore, it makes sense that leadership transformation would be a key driver of improving organisational culture.^3^

Developing self-awareness, managing oneself and continuing personal development were key aspects of the coaching process, while improving interpersonal skills was a key aspect of the CIG approach, which focused on relationship building, teamwork, networking and communication. These aspects of emotional and social intelligence may be somewhat missing from the South African competency framework for assessment of managers.^10^

Different approaches to developing leadership have been identified, such as formal training, on-the-job training, action learning and non-formal training.^6^ This study demonstrated the potential of action learning combined with on-the-job coaching and 360-degree feedback to do this successfully. A number of initiatives in South Africa have focused on more formal leadership courses and teaching, whose impact may be more limited.^6^ Action learning is a less utilised, but potentially more comprehensive approach to developing leadership.^6^

The approach to transforming organisational culture and leadership was congruent with a model of complex adaptive leadership that sees the organisation as a complex living system rather than a machine defined by reporting lines within an organogram.^11^ Complex adaptive leaders are more comfortable with uncertainty, understand the importance of connectivity and feedback within the system and the inevitability of holding paradoxical principles, for example, the need to set a number of simple rules for the organisation, while allowing people individual freedom to act within their scope of practice.

### Limitations

Members of the CIG were selected by the management without fully understanding the nature of action research. Lack of understanding their roles resulted in some frustration and participants leaving the process. Those that stayed behind benefitted from the relationships that were built and hierarchical barriers that were broken down. In hindsight, it may have been better to have taken longer to explain the process and allow departments to have more say in choosing the members. This may have led to a CIG in which people were more committed to both action and reflection. People’s sense of freedom to interact and be honest grew over time and became a strength of the group. Combining the CIG with the C^2^AIR^2^ club leaders also created some confusion and meant accommodating new members towards the end of the whole process. Transformation may have been even greater if the personal coaching had continued for longer and been extended to a broader managerial group.

The surveys were completed by different numbers of people in different proportions according to their professional roles. The follow-up survey is likely to be more accurate as the response rate is better (75/128, 59%). Although the response rate for the baseline survey is low (25/128, 20%) the results are consistent with those previously obtained for the MDHS as a whole in 2011.^8^

### Recommendations and implications

Although a substantial improvement in organisational culture was seen, the momentum needs to be maintained and this may require ongoing external facilitation. Consideration should be given as to how to take such a process to scale within an entire sub-district or district.

It should be noted that out of the five CHCs that participated in the original survey,^8^ Retreat CHC was the only one that was ready to explore change in this way. The principal researcher ensured that the facility manager and key leaders were aware of and committed to the process beforehand. This suggests that managers may be at different stages of change. Some may be pre-contemplative or unconscious of the need to change, while others may be ambivalent about the importance of change or sceptical about how to change. Exploring the current leadership style and readiness to change of managers at different facilities may be an important aspect of going to scale with this approach.

Further research should try to explore the relationship between improved organisational culture, staff engagement and satisfaction and the quality of care offered to patients. The effect on levels of staff burnout and ability to be patient-centred could also be explored.

## Conclusion

Organisational culture at Retreat CHC was transformed over a period of 18 months with a substantial decrease in cultural entropy and embodiment of positive values such as caring, sharing information, teamwork, accountability, respect, client orientation, fairness, commitment, continuous improvement and making a difference. This was achieved through transformation of the leadership style and a focus on communication and building relationships by means of CVA and feedback, 360-degree LVA, feedback and coaching and action learning in a CIG.
